# A Huygens’ surface approach to rapid characterization of peripheral nerve stimulation

**DOI:** 10.1002/mrm.28966

**Published:** 2021-08-24

**Authors:** Mathias Davids, Bastien Guerin, Lawrence L. Wald

**Affiliations:** 1 A.A. Martinos Center for Biomedical Imaging, Department of Radiology, Massachusetts General Hospital, Charlestown, Massachusetts, USA; 2 Harvard Medical School, Boston, Massachusetts, USA; 3 Computer Assisted Clinical Medicine, Medical Faculty Mannheim, Heidelberg University, Heidelberg, Germany; 4 Harvard-MIT Division of Health Sciences Technology, Cambridge, Massachusetts, USA

**Keywords:** electromagnetic field simulation, gradient coil design, magneto-stimulation thresholds, MRI safety, neurodynamic nerve model, peripheral nerve stimulation

## Abstract

**Purpose:**

Peripheral nerve stimulation (PNS) modeling has a potential role in designing and operating MRI gradient coils but requires computationally demanding simulations of electromagnetic fields and neural responses. We demonstrate compression of an electromagnetic and neurodynamic model into a single versatile PNS matrix (P-matrix) defined on an intermediary Huygens’ surface to allow fast PNS characterization of arbitrary coil geometries and body positions.

**Methods:**

The Huygens’ surface approach divides PNS prediction into an extensive precomputation phase of the electromagnetic and neurodynamic responses, which is independent of coil geometry and patient position, and a fast coil-specific linear projection step connecting this information to a specific coil geometry. We validate the Huygens’ approach by performing PNS characterizations for 21 body and head gradients and comparing them with full electromagnetic-neurodynamic modeling. We demonstrate the value of Huygens’ surface-based PNS modeling by characterizing PNS-optimized coil windings for a wide range of patient positions and poses in two body models.

**Results:**

The PNS prediction using the Huygens’ P-matrix takes less than a minute (instead of hours to days) without compromising numerical accuracy (error ≤ 0.1%) compared to the full simulation. Using this tool, we demonstrate that coils optimized for PNS at the brain landmark using a male model can also improve PNS for other imaging applications (cardiac, abdominal, pelvic, and knee imaging) in both male and female models.

**Conclusion:**

Representing PNS information on a Huygens’ surface extended the approach’s ability to assess PNS across body positions and models and test the robustness of PNS optimization in gradient design.

## 1 | INTRODUCTION

The ability to quickly assess peripheral nerve stimulation (PNS) is becoming increasingly important for assessing, optimizing, and monitoring the safety of gradient coils in MRI^1,2^ as well as drive coils in magnetic particle imaging^3,4^ or stimulation devices. All of these cases require quick prediction of whether a candidate coil and patient position will induce action potentials in the body. In the MRI gradient coil design case, identifying the stimulation location is also important to inform design choices that balance the propensity to stimulate different areas of the body. For example, if a head gradient stimulates the shoulders before the head, it might be desirable to alter the design to balance the two body parts, to increase the overall (worst-case) PNS thresholds. More generally, detailed predictions of the onset PNS thresholds can be directly incorporated into the numerical coil optimization framework as a constraint to obtain PNS optimized coil designs.^5,6^

Combining electromagnetic (EM) modeling in detailed body models followed by neurodynamic calculations to predict nerve responses to the imposed electric fields has proven successful in predicting stimulation thresholds. Early modeling approaches of Roth et al^7^ and others^8–14^ relied on simple homogeneous geometries mimicking the human body and simplified nerves to study effects such as the impact of nerve kinks on PNS thresholds. Ladenbauer et al,^15^ Laakso et al,^16^ Neufeld et al,^17,18^ and others^19–21^ embedded a small number of realistically shaped nerve fibers in more realistic heterogeneous body models to study specific applications, such as vagus nerve, sciatic nerve, or lumbar spinal cord stimulation. This significantly increased the computational complexity of these models. More recently, our group^22–24^ and others^18,25^ developed whole-body PNS modeling frameworks, consisting of detailed EM body models equipped with atlases of hundreds of nerve fibers (~1900 in our model). Using these models, PNS threshold prediction can be performed with good accuracy (error smaller than 15%) in the context of PNS characterization of MRI gradient coils and magnetic particle imaging drive coils.^22–24^

Our modeling workflow starts with simulating the E-fields induced in a detailed heterogeneous body model by the external coil and calculating the resulting electric potential changes along all nerves in the body model. We then calculate the nerve responses using either a nonlinear electric circuit-based neurodynamic model^26–28^ or a linear predictor built on the neural activating function and modified driving function,^29–32^ which we refer to as the “PNS oracle.”^24^ The PNS threshold is determined by the lowest coil current amplitude that generates an action potential in any nerve.

The PNS oracle replaces the complex titration of a nonlinear neurodynamic model with a quickly calculable linear metric that can be incorporated in the numeric gradient winding optimization.^33–35^ The PNS oracle is computed directly from the electric potential changes along the nerves, assigning a value to each segment that corresponds to the reciprocal PNS threshold. This dramatically speeds up threshold computation and guarantees a linear relationship between the PNS oracle and the electric potentials, and thus the E-fields and the coil currents. The PNS oracle contributions from all EM sources (such as disrete coils or current density basis elements) along all nerve fibers are combined in a single P-matrix that can be incorporated during the coil winding optimization as a linear constraint to obtain PNS optimized coils.^5,6^ Although useful for characterizing specific configurations, precalculation of the P-matrix is numerically intensive (up to a week of calculation) and is specific to a particular coil former geometry, body model, and patient position and orientation within the coil. Changing any of these specifics requires recomputing a new P-matrix for the new configuration.

Here, we propose a generalization of the P-matrix formulation that allows a single precomputed P-matrix defined on a Huygens’ surface lying just outside of the body model to be quickly translated to P-matrices specific for each of the coil layers in a modern gradient coil. Although precomputation of the Huygens’ P-matrix is computationally laborious (days), generation of new P-matrices for specific coil geometries or patient positions takes less than a minute. The translation step is not limited to cylinders, allowing the approach to be used to explore noncylindrical, arbitrarily shaped coil designs. If the winding pattern of a gradient is established, its fields can be quickly mapped to the Huygens’ surfaces to evaluate the PNS thresholds for multiple patient positions within these geometries. We demonstrate the value of the Huygens’ P-matrix by performing an extensive characterization of PNS-optimized gradient coils for multiple patient positions.

## 2 | METHODS

### 2.1 | Basic workflow

**FIGURE 1:**
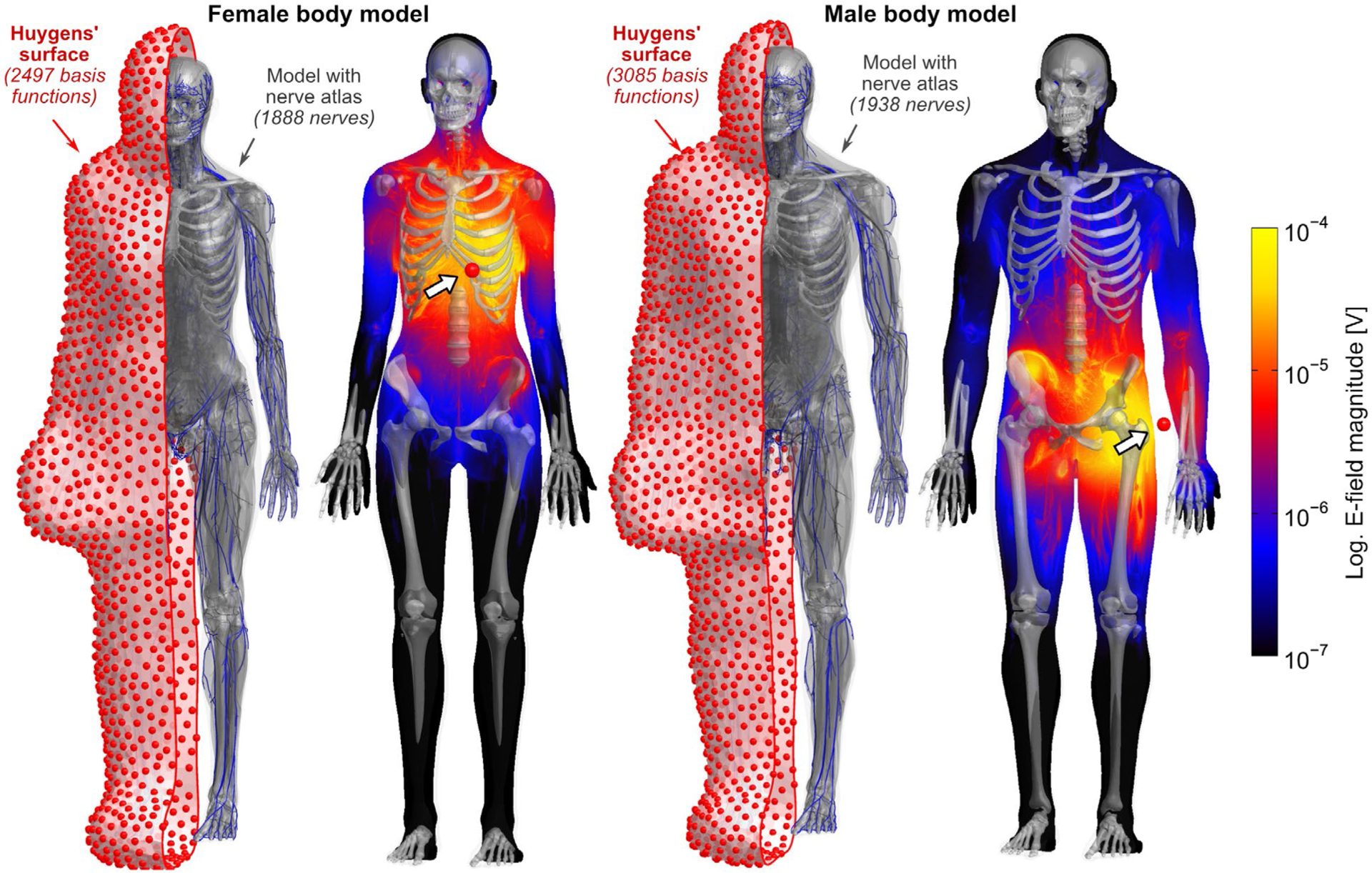
Female and male body models with enclosed Huygens’ surfaces populated with basis functions. The red dots indicate the center of each small current loop forming the basis. The E-field maps (maximum intensity projections with logarithmic scale) are shown for two exemplary Huygens’ bases near the heart and near the left arm

The Huygens’ surface consists of a mesh enclosing the entire body model at 5 cm distance to the skin (Figure 1). The surface is populated by an intermediate Huygens’ basis set (small current loops). Huygens’s principle and Green’s third identity show that these are capable of approximating the EM fields within the body model generated by any source situated outside the Huygens’ surface.^36,37^ The 5 cm surface-body distance allows the body model and its Huygens’ surface to be placed at clinically relevant patient positions within the coil (without intersecting the coil), while maintaining sufficient distance to the body to allow accurate representation of the EM fields with the finite basis set defined on the Huygens’ surface. We chose small loops of current (magnetic dipoles) with diameters 1–2 cm as the basis element. We used 2497 bases for the female model and 3085 for the male. Note that other basis elements such as electric dipoles can be added to the Huygens’ basis set to allow for modeling nonconservative E-fields such as those generated by surface electrodes. For a unit current in each Huygens’ basis we computed:

1. The B-field components at analysis points equally distributed within the body model (10 mm step size). The B-fields can be simulated quickly using the Biot-Savart formulation.
2. The E-field components at each voxel in the body model (as shown in Figure 1) using a low-frequency magneto-quasistatic solver based on the modular finite element methods (MFEM) C++ library.^38^ The E-field is sampled along each nerve in the body model and used to compute the PNS oracle values^39^ (reciprocal PNS threshold) along all nerves. This yields a PNS oracle contribution for unit excitation of a single Huygens’ basis for each 0.1 mm length nerve section. The E-field simulation and PNS oracle calculation steps are described further in the Supporting Information.

After this is done for each Huygens’ basis, the data is assembled into matrices of *n*_*H*_ columns (where *n*_*H*_ denotes the number of Huygens’ basis functions). We use the subscript “H” for matrices defined for the Huygens’ basis set. The precomputation generates the matrices ***B***_*H*_ and ***P***_*H*_ for the B-fields and PNS oracles, respectively. The matrix ***B***_*H*_ has size *n*_*B*_ × *n*_*H*_ (*n*_*B*_, number of B-field analysis points), whereas matrix ***P***_*H*_ has a size *n*_*P*_ × *n*_*H*_ (*n*_*P*_, number of 0.1 mm nerve sections in the body). Multiplication of these matrices by the vector of basis current weights describes the total B-field and PNS oracle responses, respectively.

The Huygens’ P-matrix is both body model and current-waveform specific but is independent of the coil geometry or patient position within the coil; it represents only how the Huygens’ basis set contributes to the PNS oracles. To inform a specific coil geometry and patient position, a mapping matrix is needed to link the Huygens’ P-matrix ***P***_*H*_ to the P-matrix specific to a particular gradient coil geometry, ***P***_*C*_. This is done through multiplication with an additional mapping matrix ***M*** with size *n*_*H*_ × *n*_*C*_(*n*_*C*_, number of excitation sources of the gradient coil geometry) as follows:

(1) $$P_{C}\approx {\tilde{P}}_{C}=P_{H}M$$

where ${\tilde{P}}_{C}$ is only an estimate of the desired “ground-truth” PNS oracle matrix ***P***_*C*_. A sufficient number of basis functions on the Huygens’ surface is needed to ensure that ${\tilde{P}}_{C}$ is sufficiently close to ***P***_*C*_. Importantly, the same linear relation holds for the B-fields (ie, the magnetic field ***B***_*C*_ of a set of excitation sources can be approximated by a weighted sum of the B-fields of the Huygens’ basis set):

(2) $$B_{C}\approx {\tilde{B}}_{C}=B_{H}M$$

where ***B***_*C*_ has a size of *n*_*B*_ × *n*_*C*_ (*n*_*B*_, number of analysis points in the body model; *n*_*C*_, number of excitation sources of the gradient coil geometry). Because the B-field is relatively smooth within the body model, *n*_*B*_ can be significantly smaller (we used 18 000 for our male model) than the number of mesh elements in the body model (76 million for the male model). Note that the mapping matrix, ***M***, is the same in Equations 1 and 2 due to the uniqueness of Maxwell’s equations in lossless regions.^40^ Specifically, there is a “bijective link” between B-fields and E-fields in source-free regions. Together with the well-known superposition principle for EM fields^41^ and the linearity of the PNS oracle, this ensures the same matrix can be used for both Equations 1 and 2. As mentioned previously, computing ***B***_*C*_ is not computationally intensive using the Biot-Savart formulation. We therefore use this relationship to estimate the mapping matrix ***M*** using a Tikhonov regularized pseudo inverse:

(3) $$M={\left(B_{H}^{T}B_{H}+\lambda^{2}I\right)}^{-1}\;B_{H}^{T}B_{C}$$

where ***I*** is the identity matrix and *λ* is the regularization strength (we used *λ* = 10^−7^). Generation of the mapping matrix ***M*** takes less than a minute, depending on the number and complexity of excitation sources *n*_*C*_ and the number of analysis points. On the other hand, generation of the Huygens’ ***P***_*H*_ matrix is slow (a couple of days per model), but has to be done only once per body model. The mapping matrix ***M*** must be re-evaluated for different coil geometries and different patient positions within the coil.

### 2.2 | Comparison of Huygens’ estimates to full EM calculations

We compared the B-field, E-field, and the PNS oracle values generated through Equation 3 (Huygens’ surface representation) with that from the computationally intensive direct calculation to test how well the fast Huygens’ based solution approximates the expensive full model (EM simulation plus PNS oracle extraction). We assessed the accuracy of the approach for the female and male model in seven Siemens gradient coils (Siemens Healthineers, Erlangen, Germany): four whole-body gradients (Sonata, Quantum, Prisma, and Connectome) and three head-only gradients (AC84, AC88, and the more recent “Impulse” head gradient^54^). We compared the estimates ${\tilde{B}}_{C}$, ${\tilde{E}}_{C}$, and ${\tilde{P}}_{C}$ with their directly computed counterparts ***B***_*C*_, ***E***_*C*_, and ***P***_*C*_ (computed using full EM simulations from the coil windings) as follows:

(4) $$\chi_{B}=\text{max}\left\{\frac{\left|B_{C}-{\tilde{B}}_{C}\right|}{\left|B_{C}\right|}\right\}\;\chi_{E}=\text{max}\left\{\frac{\left|E_{C}-{\tilde{E}}_{C}\right|}{\left|E_{C}\right|}\right\}\;\chi_{P}=\text{max}\left\{\frac{\left|P_{C}-{\tilde{P}}_{C}\right|}{\left|P_{C}\right|}\right\}.$$

Note that we only assessed this metric in regions where the amplitude of the quantity is above 1% of its global maximum, as the metric is ill-posed in low-amplitude regions. We assessed the error as a function of the number of basis functions on the Huygens’ surface by randomly removing basis functions and reporting the resulting error. Truncation of the full Huygens’ basis sets was done randomly, and we report *χ* for the average of 200 such choices.

### 2.3 | Assessment of PNS optimized coils at multiple patient positions

We use our Huygens’ P-matrix approach to design high-linearity, PNS-optimized, whole-body gradient coils with realistic engineering constraints using our recently described workflow.^6^ The optimization seeks a minimum inductance solution subject to a number of constraints. The coil geometry consisted of two cylindrical layers with diameters of 66 cm and 82 cm and a length of 144 cm for the primary and shield windings. The target sensitivity was set to *G*_eff_ = 0.08 mT/m per 1 A of coil current. The field linearity was constrained to 5% maximum deviation over a 40 cm diameter of spherical volume. We used the linearity definition

(5) $$\varepsilon =\frac{\text{max}\;\left\{\left|B_{\text{tar}}^{z}-B_{\text{coil}}^{z}\right|\right\}}{\text{max}\;\left\{\left|B_{\text{tar}}^{z}\right|\right\}},$$

where $B_{\text{tar}}^{z}$ tar and $B_{\text{coil}}^{z}$ coil denote the target and achieved *B*_*z*_ field in the region of linearity.^42,43^ Although not constrained during optimization, we also report the maximum fractional positional error (FPE) and the gradient uniformity (maximum pixel size error, PSE),^44^ defined as

(6) $$\text{FPE}\;(\text{r})=\frac{1}{R}\left(\frac{B_{z}(r)}{G_{\text{eff}}}-n\right)\text{ and PSE}(\text{r})=\frac{1}{G_{\text{eff}}}\frac{\partial B_{z}(r)}{\partial n}-1,$$

where R denotes the radius of the imaging volume and n is the gradient direction (x, y, or z). Maximum torque and force were constrained to 0.4 Nm/A and 0.3 N/A per axis, respectively, in the B_0_ field pattern of a realistic magnet (Siemens Prisma). All coils are actively shielded and constrained to produce a maximum B-field at the cryostat of 0.5 μT/A. A minimum wire spacing of 9 mm was enforced. PNS was constrained by computing the P-matrix for the male model for a brain imaging landmark position (head first supine) for a 250 μs rise time trapezoidal waveform with 16 bipolar pulses. We iteratively solved the coil design problem while enforcing PNS optimization in small steps (L-curve analysis), until the design problem became infeasible (no solution satisfying all constraints). A detailed explanation of this coil optimization workflow was published recently.^6^

After the design phase, we chose designs from the L-curves that allowed a 15% inductance increase compared to the unoptimized coils. The PNS performance of these coils was assessed for rise times between 100 μs and 700 μs for the male model and a brain landmark imaging position. We also analyzed these six coils for 66 body positions stepping through z-positions between −40 cm and +120 cm in 5 cm steps (z = 0 cm represents head-at-isocenter). Every position was assessed for both head-first and feet-first supine patient pose, regardless of whether the pose made sense clinically. All position calculations were done using both the male and female model. We additionally analyzed prone positions corresponding to a breast imaging landmark in the female model and a prone prostate imaging landmark in the male model.

## 3 | RESULTS

### 3.1 | E-fields and P-matrices

Figure 1 shows E-field maps (maximum intensity projection, logarithmic scale) in the female and male models for two exemplary Huygens’ basis functions: one near the heart and one near the left arm. The E-field simulations in the female model (48.8 million mesh elements) took 105 seconds per basis, with an additional 14 minutes needed for initialization (needed only once for the whole basis set). Simulation of a single basis for the male model (76.3 million mesh elements) took 170 seconds, with 37 minutes needed for initialization. The total time for computing E-fields for all basis elements was 2.5 days for the female model (2497 basis functions) and 4.5 days for the male model (3085 basis functions).

**FIGURE 2:**
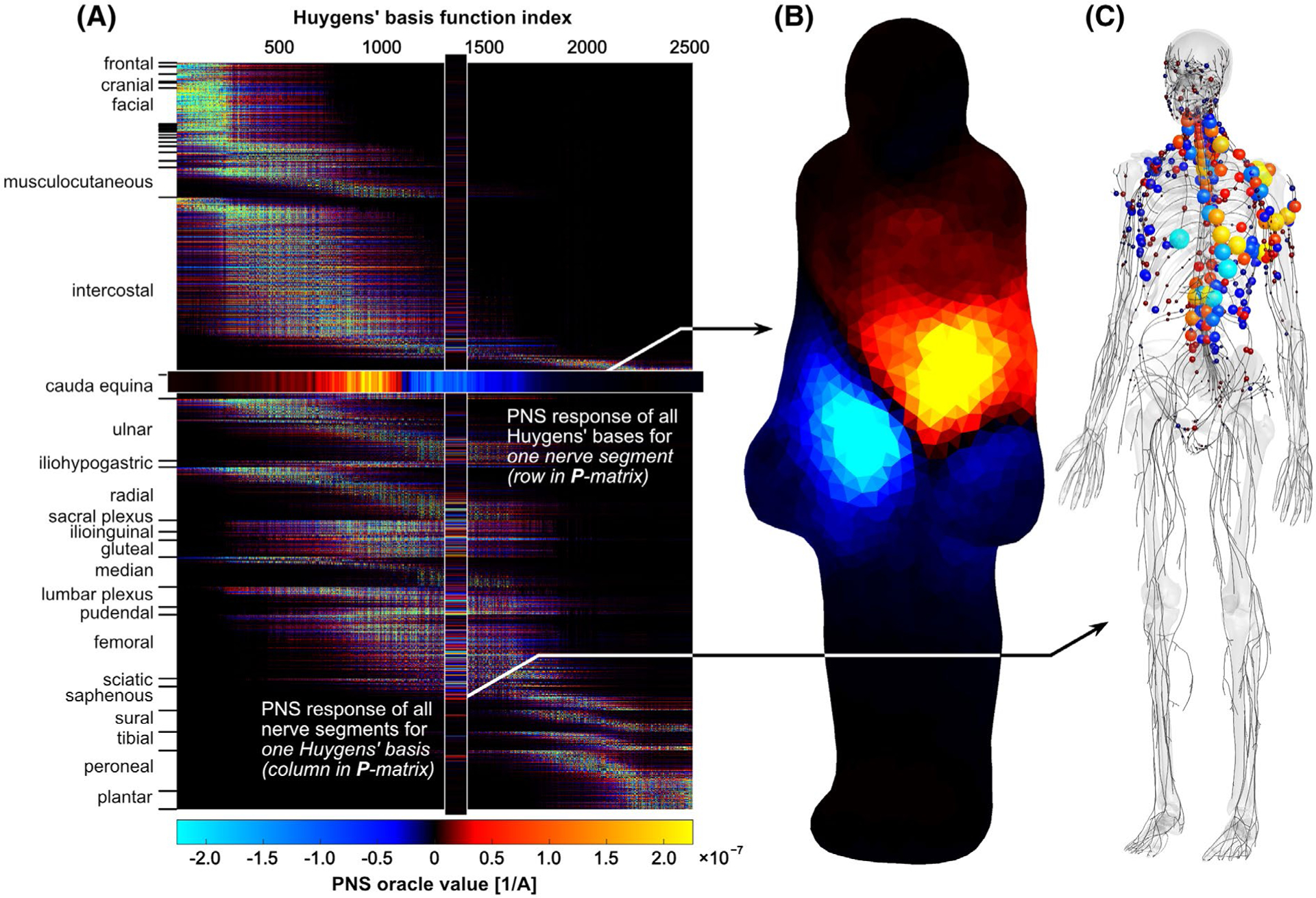
A, Huygens’ P-matrix for female body model. Each element gives the peripheral nerve stimulation (PNS) oracle value (ie, the reciprocal PNS threshold) for a 0.1 mm nerve segment (rows) and for one Huygens’ basis function (columns). The full PNS response to a given coil is obtained from a weighted sum of the basis functions associated with that gradient winding pattern and body model position. B, Plot of the PNS oracle for a single 0.1 mm segment of the cauda equina (lower end of the spine). The color mapped onto the Huygens’ surface indicates the PNS oracle contribution of each basis function. C, Plot of the PNS oracle of all nerve segments for a single Huygens’ basis function (the highlighted column in the P-matrix). The basis function examined was located anterior of the left prectoralis muscle (same Huygens’ basis as shown in Figure 1, left)

Figure 2A shows the final Huygens’ P-matrix for the female model. Both the Huygens’ basis functions (columns) and nerve groups (rows) are ordered by their z-location from head to foot. Figure 2B shows the PNS oracle responses of a single nerve segment of the cauda equina located at the lower end of the spine. The current basis elements on the Huygens’ surface are color-coded to show the amplitude of the PNS response of that nerve segment. The data in Figure 2B corresponds to a single row in the Huygens’ P-matrix. Conversely, Figure 2C shows the PNS oracle responses in all nerves induced by a single Huygens’ basis (corresponding to a single column in the Huygens’ P-matrix). The Huygens’ basis chosen in Figure 2C corresponds to the basis shown in Figure 1, left.

### 3.2 | Convergence analysis

**FIGURE 3:**
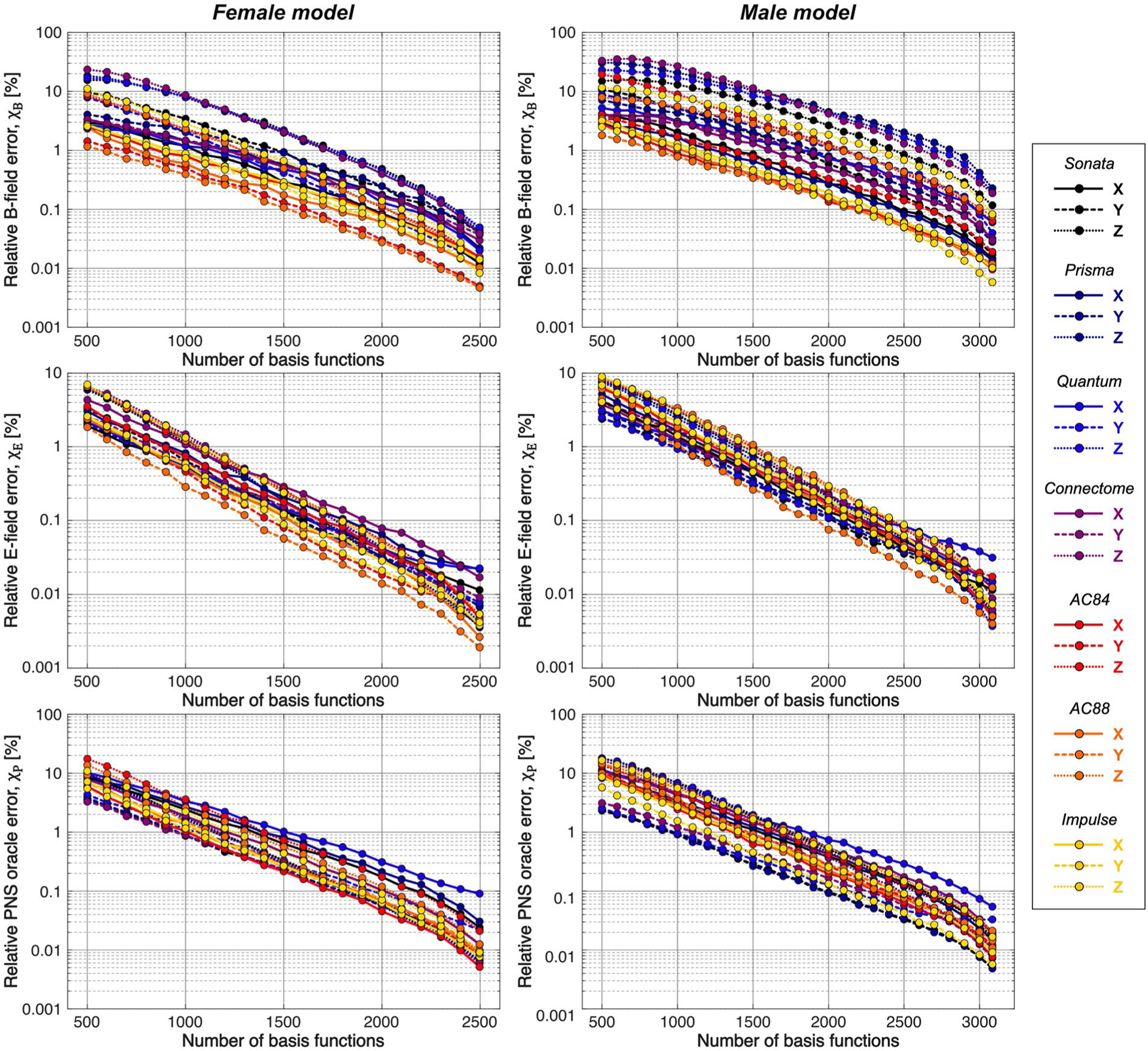
Logarithmic plot of the relative B-field error, E-field error, and PNS oracle prediction error as a function of number of Huygens’ bases for all 21 studied commercially available gradient coils in both the female model (left) and the male model (right). The coils are labeled by their Siemens marketing name. A single point denotes the average prediction error over 200 different randomly chosen Huygens’ basis sets of identical sizes

Figure 3 summarizes the convergence analysis for the Huygens’-based B-field, E-field, and PNS oracle estimates ${\tilde{B}}_{C}$, ${\tilde{E}}_{C}$, and ${\tilde{P}}_{C}$ to their directly computed counterparts (***B***_*C*_, ***E***_*C*_, and ***P***_*C*_) for the female and male model and all 21 studied coils as a function of the number of Huygens’ basis elements (*n*_*H*_). Every point corresponds to the average prediction error for 200 different randomly chosen basis sets. The convergence of the B-field error (*χ*_*B*_ defined in Equation 4) was slightly super-inear in *n*_*H*_. The full Huygens’ sets (*n*_*H*_ = 2497 or 3085 for the female and male, respectively) yielded prediction errors *χ*_*B*_ ≤ 1 % for all coils. The E-field and PNS oracle estimates converged mostly linear, reaching a prediction error of *χ*_*E*_, *χ*_*P*_ < 0.1% for all cases.

### 3.3 | PNS characterization of optimized and unoptimized Y-axis coils

**FIGURE 4:**
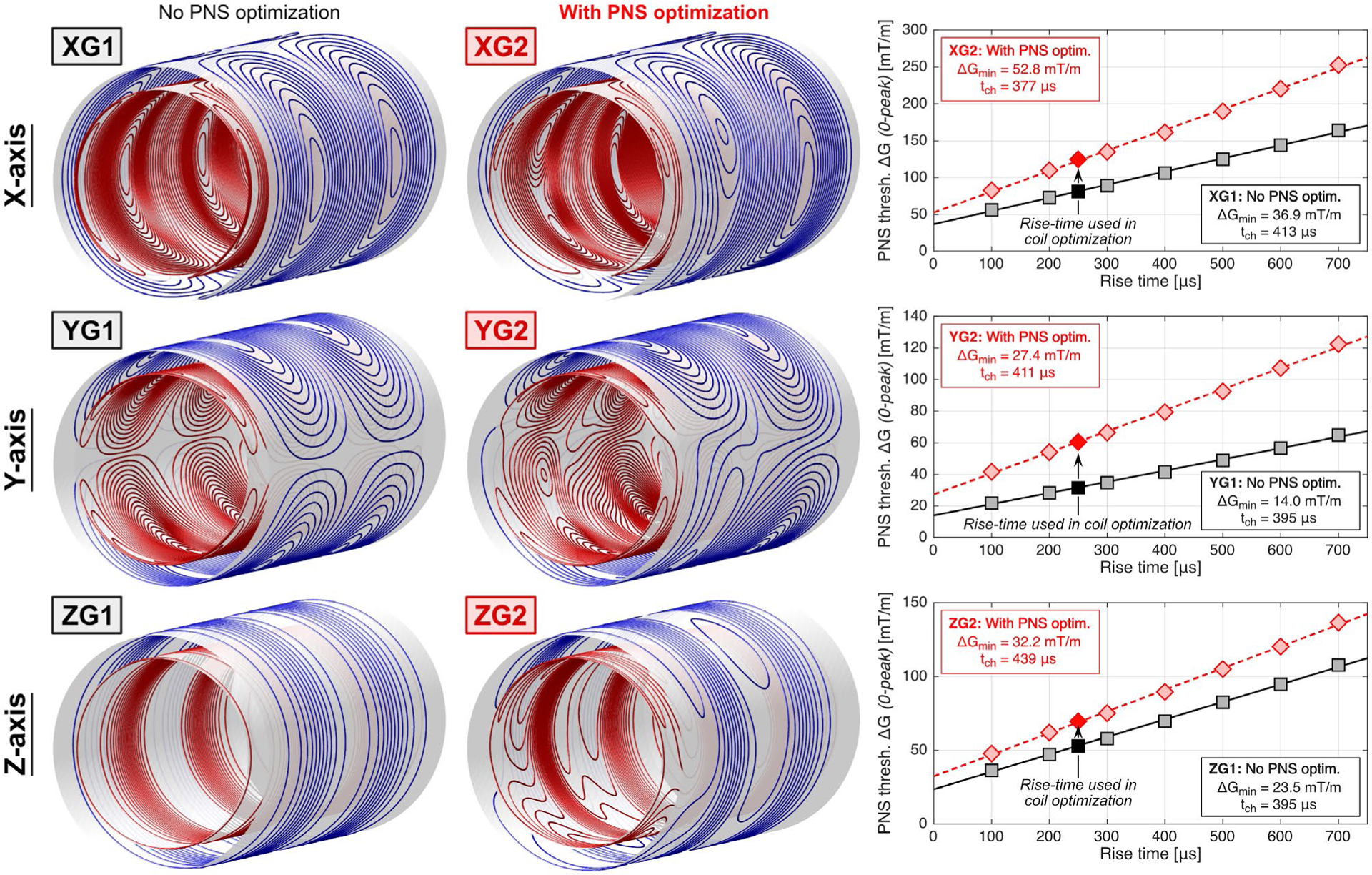
Actively shielded whole-body gradient coils without PNS optimization (left) and with PNS optimization (right). The coils were designed using a recently published PNS constrained boundary element method stream function (BEM-SF) optimization framework^6^ using the male electromagnetic (EM) and nerve body model. Optimization was performed for a head-imaging position (head-first supine body position). The increase in coil inductance for the PNS optimized coils (XG2, YG2, ZG2) was restricted to 15% compared to the coils without PNS optimization (XG1, YG1, ZG1). We also show resulting PNS threshold curves from the Huygens’ P-matrix approach for rise-times between 100 μs and 700 μs (16 bipolar pulses, 500 μs flat-top duration). Every point of the threshold curve is obtained using a waveform-specific (ie, rise-time specific) Huygens’ P-matrix. Note that the chronaxie (pulse duration at which threshold is twice the minimum threshold ΔG_min_) changes between different coil axes as seen in various experimental studies^45–48^ and between unoptimized/optimized coils

Figure 4 shows the coil winding patterns designed without PNS optimization (XG1, YG1, ZG1) and their versions with PNS optimization in the male model for head imaging (XG2, YG2, ZG2). Unfolded winding patterns are shown in Supporting Information Figure S1. The unoptimized coils correspond to typical symmetric body gradients. On the contrary, PNS optimization breaks the typical coil symmetries. These modifications increased PNS thresholds by 54%, 92%, and 31% (for the X, Y, and Z axes), while increasing the coil inductance by 15%. A detailed summary of all engineering metrics of these six coils is shown in Table 1. Figure 4 also shows PNS threshold curves generated using the Huygens’ P-matrix formalism for a wider range of rise times between 100 μs and 700 μs, while the winding optimization itself only used a P-matrix constraint corresponding to a 250 μs rise time. Note that the chronaxie, ie, the pulse duration (twice rise-time) at which the PNS threshold is twice the minimum threshold ΔG_min_ is different for different coil axes (as is well known from experiments^45–48^), but also changed between unoptimized and optimized coil layouts. A summary of all PNS threshold curve parameters, including ΔG_min_ and SR_min_ parameters,^60^ is given in Supporting Information Table S1.

**FIGURE 5:**
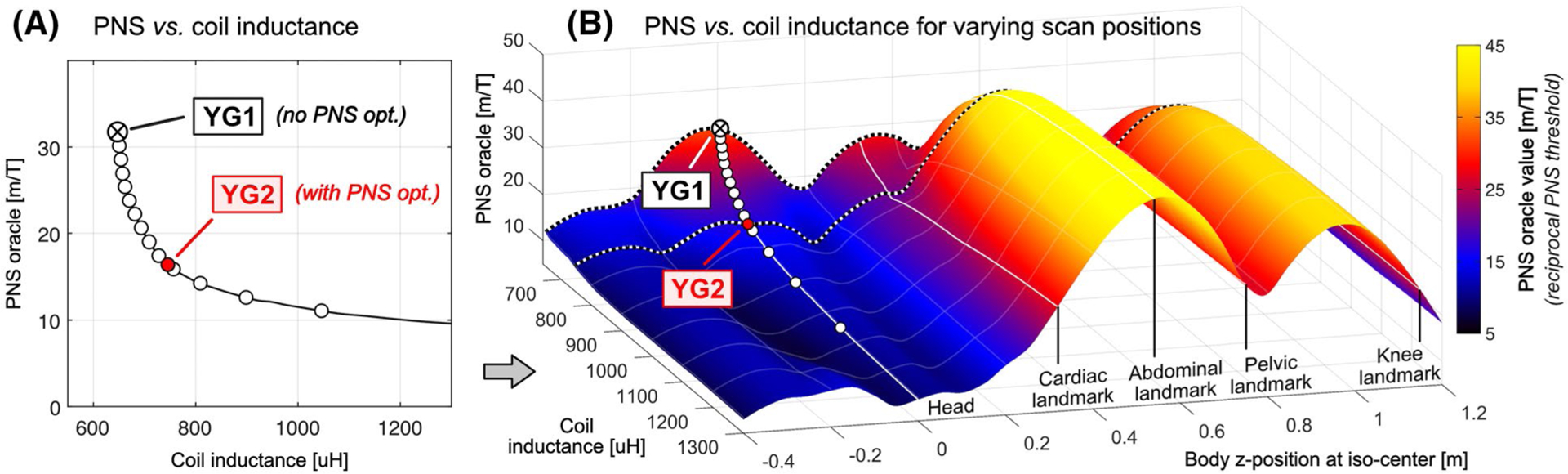
A, L-curve for optimized actively-shielded Y-axis gradient coils showing tradeoff between PNS oracle (reciprocal PNS threshold) and coil inductance. The coils were optimized for the male body model for head imaging with head-first supine orientation. Other constraints such as gradient sensitivity, field linearity, torque, and minimum wire spacing were kept constant (see Table 1). Every point on the L-curve corresponds to a coil winding solution (two solutions marked YG1 and YG2 are shown in Figure 4). Coil solution YG1 is a design generated from a conventional optimization (no PNS constraint). In solution YG2, the PNS constraint generated a design with a higher PNS threshold, but higher coil inductance. B, To assess the PNS performance of solutions YG1 and YG2 for non-head imaging, the PNS performance of the coil-winding patterns found in (A) were reassessed with the patient at different positions along the z-axis of the coil using the Huygens’ surface representation. The resulting surface shows the PNS-inductance tradeoff when the coils are used at every body position. The PNS thresholds for coils YG1 and YG2 as a function of body position are shown by dotted lines. Using a P-matrix linked to a Huygens’ basis set enables this analysis, as this P-matrix is a body-position invariant

Figure 5A shows an L-curve analysis of the tradeoff between the maximum PNS oracle (reciprocal PNS threshold) as a function of the coil inductance for whole-body Y-axis gradient coils optimized for the male body model for head imaging (head-first supine). Every point on the L-curve corresponds to a coil winding solution, including the designs YG1 and YG2 shown in Figure 4.

Figure 5B extends the PNS analysis to show how the coils on the L-curve of Figure 5A perform for other patient positions. The additional dimension corresponds to the z-position of the male body in the coils. Some of the z-positions mimicking clinically relevant scan positions are marked by solid lines (namely head, cardiac, abdominal, pelvic, and knee imaging). Dotted lines are used to trace the PNS oracle behavior of the coil solutions YG1 and YG2 for the varying body z-positions. Note that the L-curve data in Figure 5A correspond to a single line in the surface of Figure 5B.

Optimization of the coil windings for head imaging (head-first supine) led to PNS oracle improvements for that body position of 48% (92% increased thresholds, YG2 compared to YG1) and improved the PNS characteristics for cardiac imaging, although to a lesser degree (23% PNS oracle reduction, 29% increased PNS threshold). For the body position mimicking abdominal, pelvic and knee imaging, however, coil solution YG2 had degraded PNS performance compared to the unoptimized coil (PNS oracle increases of +21%, +15%, and +15%; PNS thresholds decreases of −26%, −17%, and −18%, respectively).

**FIGURE 6:**
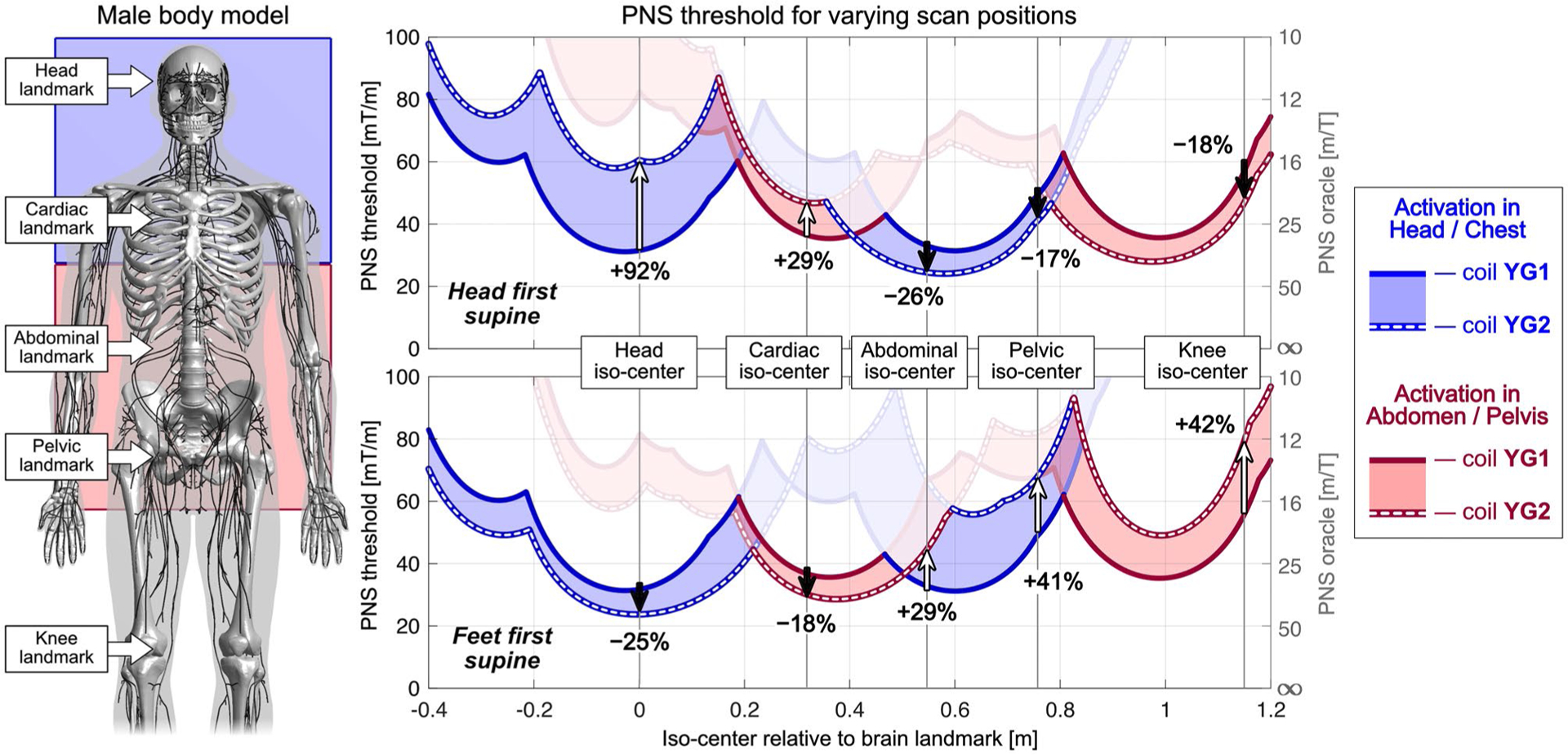
Analysis of the two dotted curves (coil designs YG1 and YG2) of Figure 5, displayed as PNS thresholds as a function of body position of the male model. This describes how the 92% PNS threshold improvement of coil YG1 (which was optimized on the male model for head at iso-center, head-first supine) changes for other body positions. The color of the curve refers to the location of the activation. Regions where the dashed line (PNS constrained coil YG2 thresholds) lies above the solid line (non-PNS constrained coil YG1) indicates use-cases that retain some benefit from the optimized design, even though the optimization only considered a head-at-isocenter body position. The curves with the worst-case threshold are highlighted in non-transparent coloring. For head-first supine patient positions (top graph) typically used for head and cardiac imaging, coil YG2 was beneficial in both cases (+92% and +29% threshold changes, respectively). For abdominal, pelvic and knee imaging, which would typically be done with a feet-first supine position (bottom curves), the optimized design raised thresholds by +29%, +41%, and +42%, respectively, when the patient was inserted in the feet-first supine body position

Figure 6 shows a more detailed analysis of the PNS threshold characteristics of the unoptimized coil YG1 (solid curve) and optimized coil YG2 (dashed curve) for varying positions of the male model and for both head-first and feet-first orientations. Regions where the dashed line lies above the solid line indicate use-cases that retain some benefit from the PNS optimized design even though the optimization was performed for head-at-isocenter. The color corresponds to the origin of the nerve activation in the body: Blue denotes first activation in the head/chest, and red denotes activation in the abdomen/pelvis.

As seen in the L-surface analysis in Figure 5, the PNS-optimized coil YG2 achieves higher PNS thresholds than the unoptimized coil YG1 for head-first supine orientation and for head and cardiac imaging. The origin of PNS changes from head/chest (blue region of interest) to an abdomen/pelvis activation site (red region of interest) after a body position of approximately 0.2 m relative to the head landmark. For relative body positions greater than 0.4 m, corresponding to landmarks for abdominal, pelvic, or knee imaging, coil YG2 led to lower PNS thresholds (degraded performance) compared to coil YG1. Figure 6 (bottom) also shows the corresponding results for a feet-first supine orientation. For the unoptimized symmetric coil YG1, changing the body orientation from head-first to feet-first does not affect the PNS threshold characteristics. For coil YG2, using feet-first rather than a head-first orientation degraded PNS performance for head and cardiac imaging (threshold changes of −25% and −18%). For abdominal, pelvic and knee imaging, moving to feet-first orientations improved PNS performance by +29%, +41%, and +42%, respectively.

**FIGURE 7:**
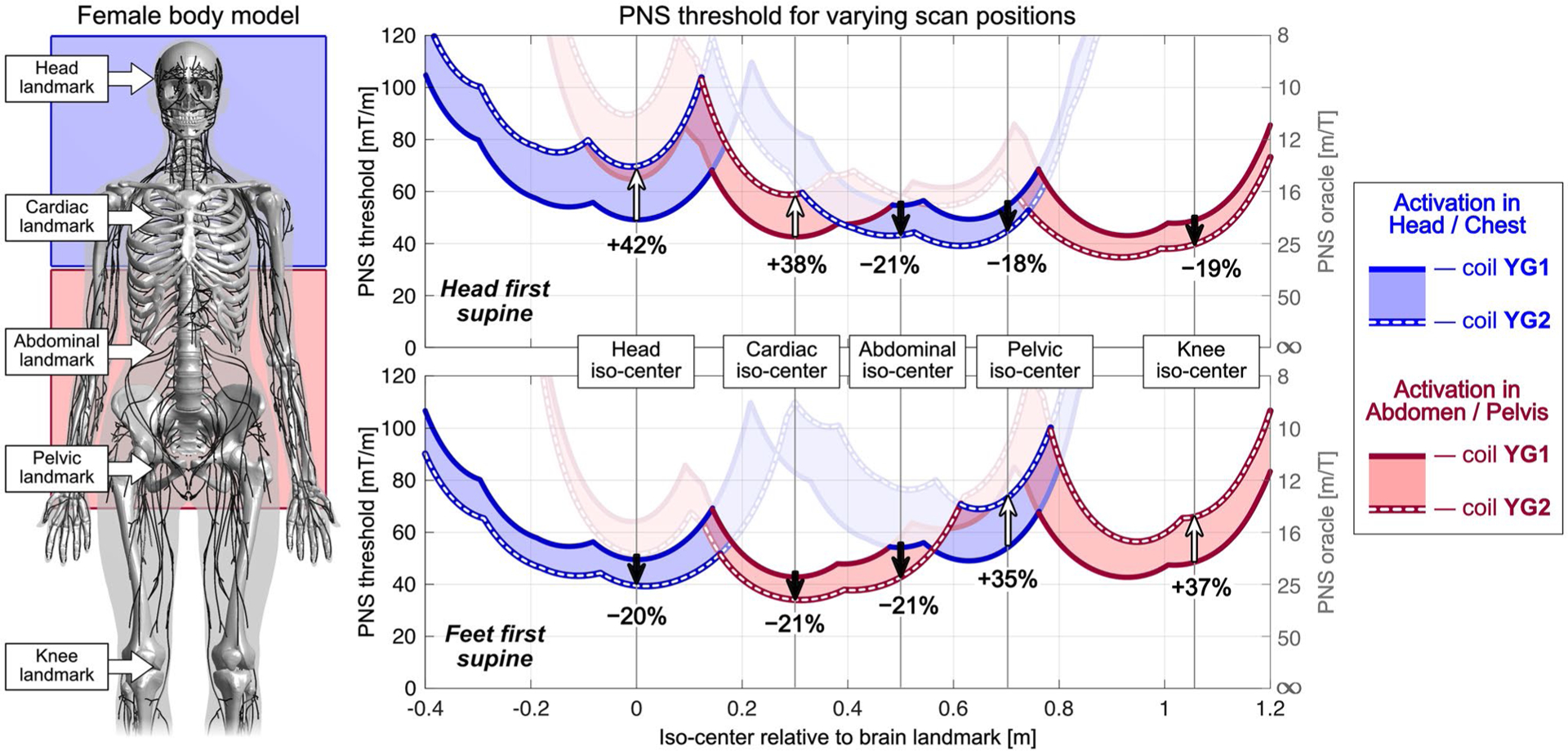
Results from the female model for the analysis shown in Figure 6. Use cases in which the optimized coil YG2 coil yields higher PNS thresholds than coil YG1 are similar to the results in the male model (except for abdominal imaging): PNS thresholds increase for head-first supine head and cardiac imaging (top graph) and for feet-first supine pelvic and knee imaging (bottom graph)

Figure 7 shows a similar PNS characterization for coils YG1 and YG2 for the female model. Here, using the optimized coil YG2 in a head-first body orientation (top panel) showed improved PNS thresholds for head and cardiac imaging (+42% and +38%, respectively), but degraded PNS performance for abdominal, pelvic, and knee imaging (thresholds changes of −21%, −18%, and −19%). These results are qualitatively similar to those found in the male model, although PNS thresholds varied more strongly in the male model when changing the body position.

### 3.4 | Male model activation maps

**FIGURE 8:**
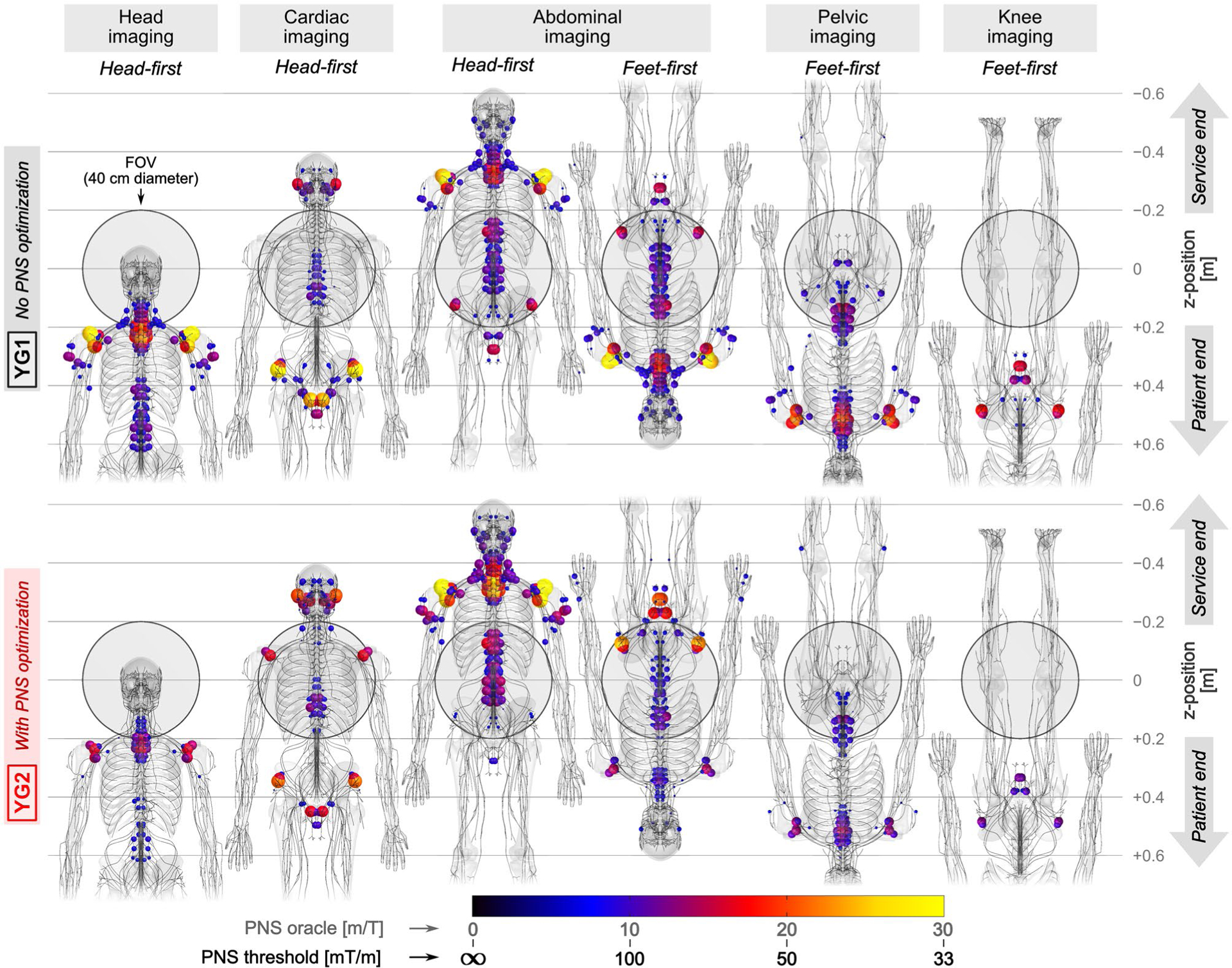
PNS activation in the male model expected for the different body positions in the coils YG1 (top) and YG2 (bottom). The gray shaded circle denotes the 40 cm region of 5% gradient linearity. Colored spheres show hotspots with largest PNS oracle values (smallest PNS threshold). For the abdominal imaging position, we show both head-first and feet-first supine positions; all other scan positions use either head-first (head and cardiac imaging) or feet-first position (pelvic and knee imaging). In all cases shown, meant to correspond to conventional clinical patient positions, the optimized coil retains some value in raising the PNS threshold (lowered PNS oracle), except for the head-first supine abdominal imaging case

Figure 8 shows PNS hot-spot maps (locations of strongest activation) in the male model for both YG1 and YG2 coils. Columns correspond to different scan positions and body orientations, namely, head imaging (head-first orientation), cardiac imaging (head-first), abdominal imaging (both head-first and feet-first), as well as pelvic imaging and knee imaging (both feet-first). The distribution of activation patterns was similar in many cases, with typical activation sites in the shoulders, upper arms, intercostal nerves, neck, and pelvic area nerves, as well as nerves close to the ears and jaws. In all cases except for head-first abdominal imaging, the optimized coil YG2 improved PNS performance (lower PNS oracle, increased PNS thresholds) compared to the unoptimized coil YG1, even though this coil was only optimized for head-first supine head imaging.

### 3.5 | PNS changes in all gradient axes

**FIGURE 9:**
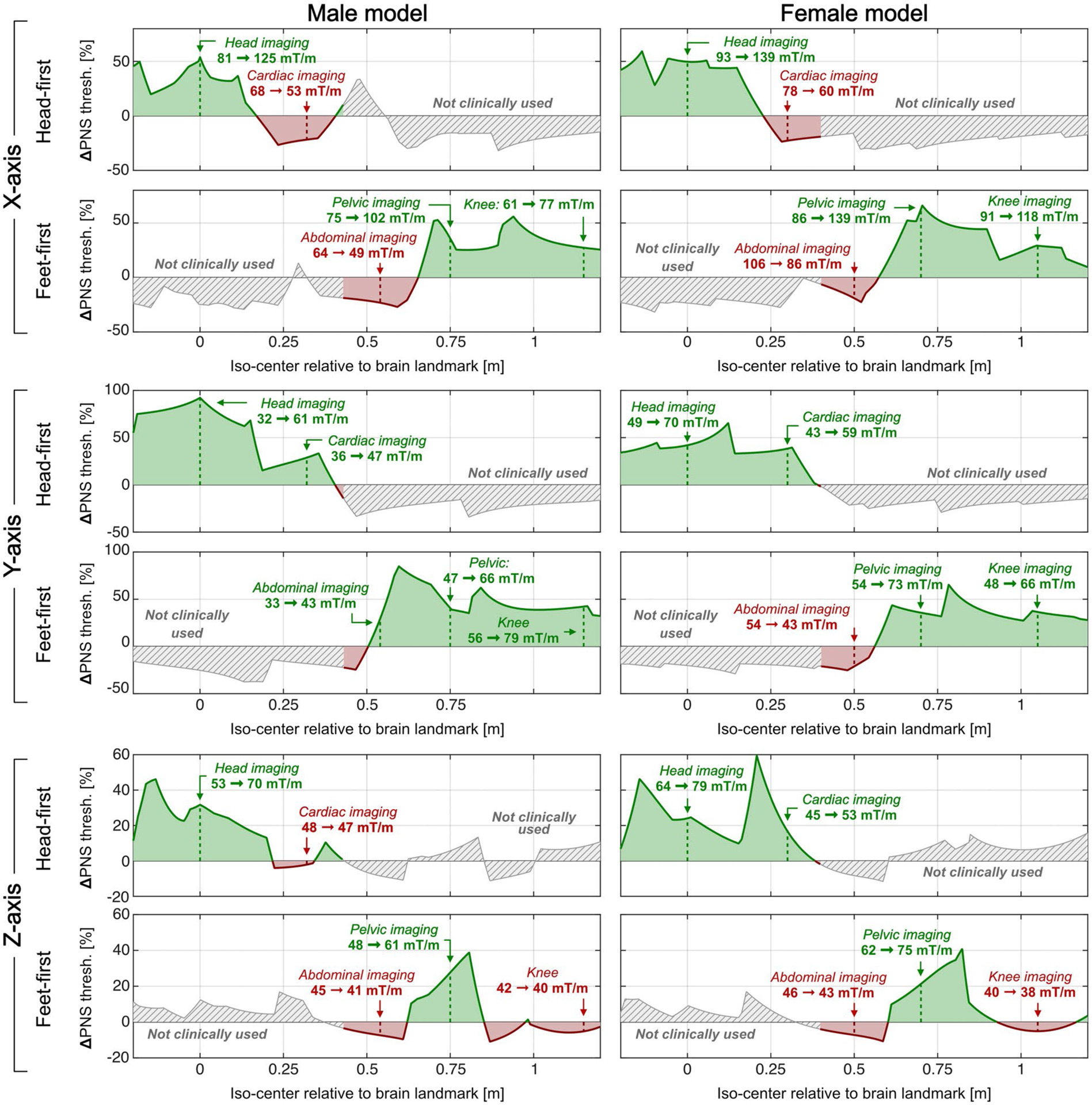
Summary of percentage PNS threshold changes as a function of scan position (iso-orientation (head-first and feet-first) for the X, Y, and Z-axes coils in both the male and female model (left and right columns). All PNS thresholds are based on a trapezoidal coil waveform (250 μs rise time, 500 μs flat-top, 16 bipolar pulses). We also report quantitative PNS threshold changes for the five clinically relevant scan positions studied in this work. Note that PNS optimization of the X-axis coil for head imaging led to reduced PNS thresholds for cardiac imaging. PNS optimization of the Z-axis coil slightly reduced PNS thresholds for abdominal and knee imaging (< 7% in both cases). The average PNS threshold changes were +24%, +40%, and +9% for the X, Y, and Z-axes coils, respectively (averaged over all studied clinical scan positions in both models)

Figure 9 summarizes the percentage PNS threshold changes from the optimization as a function of scan position (iso-center relative to brain landmark) and body orientation (head-first and feet-first) for all coil axes and both body models. Additionally, we report quantitative PNS threshold changes for the five clinically relevant scan positions. PNS optimization of the X-axis coil for head imaging reduced PNS thresholds for cardiac imaging by −21% to −24%. PNS optimization of the Z-axis coil slightly reduced PNS thresholds for abdominal and knee imaging (< 7% in both cases). The average PNS threshold changes were +24%, +40%, and +9% for the X, Y, and Z-axes coils, respectively (averaged over all studied clinical scan positions in both models). We also analyzed a head-first prone orientation at a breast imaging position (female model only) and found threshold changes of −28%, +43%, and +23% (for the X, Y, and Z-axes coils). A similar analysis for a feet-first prone position at a prostate orientation (male model only) led to threshold changes of +12%, +22%, and −4% (for the X, Y, and Z-axes coils).

## 4 | DISCUSSION

We present and validate a fast PNS modeling approach based on a linear PNS matrix (P-matrix) defined for a current basis set on a Huygens’ surface closely fitting the body model. This versatile P-matrix can be mapped rapidly to arbitrary coil winding patterns or coil former geometries, yielding a coil-specific P-matrix that fully characterizes the coil’s PNS properties (thresholds and locations). Furthermore, assessing a new patient position within the coil requires only a fast recalculation of a mapping matrix linking the two basis sets. The coil-specific P-matrix generated using the Huygens’ workflow can be readily incorporated in standard approaches for numeric winding optimization to constrain PNS. Although generation of the Huygens’ P-matrix requires substantial precomputation (multiple days per body model), once this is done, mapping to a coil-specific and patient position–specific P-matrix takes less than a minute. Using this tool, we demonstrated that winding patterns optimized for a single brain imaging landmark can also yield favorable PNS characteristics for many, but not all clinically relevant scan positions. For example, analysis of the head-optimized X-axis coil provided improvements for head, pelvic, and knee imaging but not for cardiac or abdominal imaging. Improvements for all possible scan positions might be difficult to achieve without enforced constraints on all scan positions.

Using the Huygens’ surface as an intermediate step in the PNS prediction allows for a dramatic simplification and speed-up of PNS modeling. This speed-up is achieved by dividing the prediction process into a computationally expensive precomputation and an efficient change-of-basis projection step. The one-time precomputation phase includes time-consuming finite element method EM field simulations in a specific detailed body model, followed by extraction of PNS responses through calculation of the linear PNS oracle along all nerves in the body model. The one-time precomputation of the Huygens’ P-matrix takes multiple days per model, but generates data independent of the coil geometry or the body position within the coil (a more detailed discussion on the overall computational complexity is given in the Supporting Information). The conversion of the PNS model into a single versatile Huygens’ P-matrix (which is constructed without any knowledge of a gradient coil geometry) also simplifies dissemination of the PNS modeling method since the Huygens’ P-matrix can be shared without the need for communicating specific wire patterns or geometries, third-party EM simulation software or a possibly proprietary body model. Instead, using the Huygens’ P-matrix (~3 GB) requires only simple Biot-Savart calculations of the B-field and solving a relatively small system of equations to obtain the mapping matrix, both of which can be easily performed using standard tools such as *MATLAB* (MathWorks, Natick, MA) or *Python* (Python Software Foundation, Wilmington, DE). Using this Huygens’ P-matrix, coil designers can generate coil former-specific P-matrices that can be introduced in the coil design algorithm to generate PNS-optimized coil solutions for any body position and former geometry (including noncylindrical geometries). Future work might also allow optimization of the coil geometry itself, as well as the winding patterns.

The fast PNS Huygens’ model allowed us to extend PNS characterization of gradient coils, which would be computationally prohibitive using full PNS modeling. The coils studied in this work were optimized specifically for our male model and for head imaging in a head-first supine orientation. The analysis suggests that PNS-optimized gradient coils can provide PNS benefits in a wide range of clinical imaging applications and in different body models. This robustness of PNS reduction at multiple patient positions likely results from the fact that the winding optimizer typically achieves PNS optimization by affecting the E-fields at the nerve locations by altering the concomitant B-field amplitudes. In the examples studied, this was accompanied by a reduced B-field and E-field region at the patient end, and increased B-fields and E-fields toward the scanner’s service end. Note that a direct application of the concomitant field strategy was introduced by Hidalgo-Tobon using an additional concomitant field coil.^49^ As long as the largest body part is placed in the reduced-field region, the PNS-optimized coil layout retains some of its benefits compared to an unoptimized design.

The Huygens’ PNS framework allows us to quickly generate various P-matrices corresponding to different positions of the body models within the coil geometries and to different waveforms. For example, including multiple P-matrices specific to different scan positions in the winding optimization allows us to simultaneously control PNS characteristics for multiple imaging modalities. We demonstrated this concept recently^6^ by jointly optimizing body Y-axis gradient coils for head and cardiac imaging. This led to more consistent, but overall lower PNS threshold improvements for these two imaging applications. Although the approach is little-tested and not validated experimentally, it could be applied to the X-axis coils analyzed in this work to eliminate or reduce the PNS threshold penalty for cardiac imaging (likely at the cost of increased coil inductance or overall reduced PNS gains). A more complete picture of the PNS thresholds can be obtained by generating at least two P-matrices at differing rise times to characterize the full linear PNS threshold curve as a function of rise time for that class of waveforms. This allows PNS constraints to be applied at multiple rise times, such as to avoid creating a coil that is better for some rise times but worse for others (eg, if the PNS threshold curves shown in Figure 4 were to cross due to differing chronaxie times). Note that the chronaxie has been observed to vary dramatically between 285 μs and 879 μs^1,23,45,46,48,50–54^ and is affected by the type of nerve^55^ and the shape of the E-field stimulus.^56^ A change in nerve chronaxie as a result of winding modifications would represent a more complicated PNS relationship between unoptimized and optimized coils (although still fully characterized). With P-matrices at two different rise times, theoptimization could be driven by the complete PNS characterization, and a cost-function or constraint could be devised according to the design goals, although this approach is untested at this time.

The linear and “signed” (positive or negative) nature of the PNS oracle formalism plays a central role in the P-matrix and Huygens’ surface–based PNS tool. The PNS oracle assigns a value to each nerve section (sampled at 0.1 mm) that describes the degree of membrane hyperpolarization and depolarization expected in response to the applied gradient waveform, such that the inverse of its magnitude yields the PNS threshold for that waveform. Although the threshold is given by the inverse of its absolute value, the oracle sign allows PNS contributions from independent coils (or current basis elements) to cancel each other. The signed and linear nature of the PNS oracle allows the use of Huygens’ principle. Alternatively, the Huygens’ approach to PNS analysis could focus solely on the E-fields rather than the PNS oracle. For example, recent works by Roemer et al^44,53^ used fast surface E-field metrics computed in homogeneous body models as a surrogate for PNS thresholds. These E-field metrics can then be incorporated in the winding optimization as an additional constraint, similarly to our PNS-constrained winding optimization.^6^ Linking E-field metrics to PNS thresholds was achieved using *a priori* estimates of the nerve chronaxie (an IEC mandated value of 360 μs for body coils, or an adjusted value of 669 μs for head coils). However, gradient coil chronaxie times have been reported ranging between 351 μs and 770 μs for whole-body gradients^1,23,45,46,48,50–52^ and between 285 μs and 879 μs for head-only gradients.^23,48,53,54^ Our PNS model requires a different sort of *a priori* parameters: those related to the nerve cable model, which are independent of the coil geometry. Additionally, the nerve model approach attempts to achieve specificity to the component of the E-field contributing to local stimulation (ie, the component along the large nerves), although this requires reliance on the model having realistic nerve paths.

Our Huygens’ P-matrices are body model and current waveform–specific. Modifying the body model by adding anatomical features or changing dielectric properties requires recomputation of the E-fields for all Huygens’ bases (taking a couple of days) and re-extraction of the waveform-specific PNS oracle values along all nerves (taking ~10 minutes per model). In contrast, generating a P-matrix for a different current waveform without modifying the body model allows reuse of the precomputed E-fields, as they can be linearly scaled to the waveforms in the magneto quasistatic regime. For example, a typical analysis of the charge-duration curve,^57,58^ more commonly expressed as gradient amplitude at threshold as a function of rise time by the MRI community,^51,59,60^ would require one P-matrix per rise time per body model.

The Huygens’ surface must fit inside any coil former under evaluation and balance proximity to body and coil with the number of basis functions needed to accurately describe the EM fields and the P-matrix. For example, a small-surface gradient coil placed close to the body would likely require a Huygens’ surface more densely populated with basis functions than those studied in this work. Fortunately, the B-field error *χ*_*B*_ can indicate whether a Huygens’ basis set is appropriate for a given coil geometry. Additionally, the PNS prediction accuracy is expected to degrade for non-divergence-free sources, such as nonclosed coils, as the basis set consisted only of divergence-free elements (current loops). The method can be supplemented with other basis function types such as electric dipoles, such as to allow modeling stimulation using surface electrodes.

The Huygens’ P-matrix approach shares the same limitations as previously introduced neurodynamic modeling based PNS simulations. The PNS oracle itself introduces a prediction error of up to about 7% compared to thresholds from numerical titration of the full neurodynamic model. Other sources of error arise from uncertainties in dielectric tissue properties, anatomical features (impacting formation of local E-field hotspots), and numerical limitations, such as stair-casing effects typical for hexahedral finite element method simulations. The nerve atlases themselves are limited, and many of the labeled axon diameters (affecting each nerve’s excitability) are indirect estimates from conduction velocity studies.^61^ Finally, of course, it is difficult to expect two body models to represent the full range of human variability or expect simulations to replace experimental measurements in the regulatory process without considerable validation. The difficulty of generating additional models is a limitation of our work, although such models can be developed from cryosections^62^ or can be generated using anatomically correct morphing algorithms.^63^ On the other hand, we have validated our PNS modeling tools by comparing predicted thresholds to experimental data obtained using multiple body and head gradient coils, and found that experimental average thresholds were well represented by the mean of the male and female model (~15% prediction accuracy).^22–24,54^ This validation includes the recent Siemens “Impulse” head gradient^54^: a high-linearity, high-performance (G_max_ = 200 mT/m, S_max_ = 900 T/m/s) gradient coil whose design phase was informed by PNS modeling to maximize PNS thresholds. Because simulation tools based on a small number of realistic body models cannot represent the range of PNS responses, they will likely first be used as a method to understand the “where” and “why” questions surrounding PNS with experimental threshold measurements used for validation and to set safe-use limits for a coil. The Huygens’ approach allows the evaluation of considerably more body positions than could be acquired experimentally and could therefore be considered to augment the experimental studies. A second goal of PNS modeling is design characterization and coil optimization. For this, a small number of models is also a hindrance, but we do not necessarily require the modeling to capture the range of human variability. We pursue a reduced goal that only requires the model to characterize the mean of the population, not the range of individual responses. In this case, we seek to raise the thresholds for the mean of the population, but not every individual or specific individuals.

Of course, additional work is required in analyzing our PNS optimized coils and their behavior in a realistic MRI environment. This includes effects of increased concomitant fields in the FOV, vibration and acoustic properties,^64,65^ secondary effects of cryostat eddy currents,^66,67^ properties of power and heat dissipation,^68,69^ and studying coil layer configurations and permutations between the ordering of the layers and their effect on PNS. The PNS optimization shown produces asymmetric winding patterns that will likely lead to asymmetric eddy currents. Although asymmetric coils are becoming more common (especially for head gradients), assessment of the effects of the asymmetry need to be further analyzed and potentially corrected for Refs. [70–74]. Additionally, the manufacturability of our optimized coil winding pattern needs to be analyzed, and further constraints might be needed to improve manufacturability (eg, by enforcing certain coil symmetries).

## 5 | CONCLUSIONS

We demonstrate compression of a combined EM and neurodynamic PNS model applicable to multiple coil geometries and patient positions into a single versatile PNS matrix (P-matrix) defined for a Huygens’ surface tightly fitting the body model. After precomputation of this Huygens’ P-matrix, it can be used to generate P-matrices specific to gradient coil geometries and patient positions within a couple of seconds. The Huygens’ P-matrix is unique to the body model and the gradient waveform used in its calculation. We use the approach to design PNS-optimized gradient coils and study their robustness with respect to patient position and orientation. The fast Huygens’ formalism greatly extends the usability of the PNS model and simplifies dissemination of simulation-based threshold prediction tools. Once the Huygens’ P-matrix is generated, it can be used without the need for EM modeling software or dissemination of a proprietary body model.

## Supplementary Material

Supplemental_figures **FIGURE S1** Unfolded winding patterns for the unoptimized and optimized body gradient coils. The coils were designed using our peripheral nerve stimulation (PNS)–constrained boundary element method–stream function (BEM-SF) optimization framework published recently^6^ and were optimized using our male body model for head-imaging using a head-first supine body position. The increase in coil inductance for the PNS-optimized coils (XG2, YG2, ZG2) was restricted to 15% compared to the coils without PNS optimization (XG1, YG1, ZG1)
**TABLE S1** Summary of PNS threshold curve parameters ΔG_min_(0-p) (minimum stimulating waveform zero-to-peak gradient amplitude), t_chron_ (chronaxie time), as well as ΔG_min_(p-p) (minimum stimulating waveform peak-to-peak gradient excursion) and SR_min_ (minimum slew-rate needed to achieve stimulation). Note: The PNS threshold curves were obtained by simulation of trapezoidal waveforms with 500-μs plateau time and 16 bipolar cycles

## Figures and Tables

**TABLE 1:** Summary of engineering and PNS metrics for the six gradient coils designed and analyzed in this work. All metrics except the fractional positional error and uniformity (pixel size error) were explicitly included in the coil winding optimization (either as a constraint or cost function). Torque and force properties were calculated based on a realistic 3T Siemens Prisma magnet (realistic magnets are often inhomogeneous in the region occupied by the gradient windings, rendering torque/force balancing more challenging). Accepting a 15% inductance penalty allowed for an up to 92% increase in PNS threshold in the male model (Y-axis)

	X-axis			Y-axis			Z-axis		
	No PNS optim. (XG1)	With PNS optim. (XG2)	Change	No PNS optim. (YG1)	With PNS optim. (YG2)	Change	No PNS optim. (ZG1)	With PNS optim. (ZG2)	Change
Inductance (μH)	618.6	710.3	+15%	647.1	742.8	+15%	326.3	375.5	+15%
PNS threshold (mT/m)*	81.1	125.0	+54%	31.5	61.0	+92%	52.8	70.0	+31%
Sensitivity (mT/m/A)	0.08	0.08	–	0.08	0.08	–	0.08	0.08	–
Linearity (%)	5.0	5.0	–	5.0	5.0	–	5.0	5.0	–
Fractional positional error (%)	5.0	5.0	–	5.0	5.0	–	5.0	5.0	–
Pixel size error (%)	27.2	27.5	+ 1%	27.5	31.6	+ 15%	29.6	30.4	+3%
Torque (Nm/A)	0.3	0.3	–	0.3	0.3	–	0.0	0.3	N/A
Force (N/A)	0.3	0.4	+33%	0.4	0.4	–	0.0	0.3	N/A
Maximum stray field at cryostat (μT/A)	0.5	0.5	–	0.5	0.5	–	0.5	0.5	–
Minimum wire spacing (mm)	9.0	9.0	–	9.0	9.0	–	11.9	9.0	−24%

Note:

* Male model, head iso-center, head-first supine, 250 μs rise time.
