# Supplementary material for: A Huygens’ surface approach to rapid characterization of peripheral nerve stimulation
Source: Magn Reson Med. Author manuscript; Available in PMC 2022 Jan 1. (PMC8689355; doi:10.1002/mrm.28966)
Supplement: Supplemental_figures — FIGURE S1 Unfolded winding patterns for the unoptimized and optimized body gradient coils. The coils were designed using our peripheral nerve stimulation (PNS)–constrained boundary element method–stream function (BEM-SF) optimization framework published recently6 and were optimized using our male body model for head-imaging using a head-first supine body position. The increase in coil inductance for the PNS-optimized coils (XG2, YG2, ZG2) was restricted to 15% compared to the coils without PNS optimization (XG1, YG1, ZG1) TABLE S1 Summary of PNS threshold curve parameters ΔGmin(0-p) (minimum stimulating waveform zero-to-peak gradient amplitude), tchron (chronaxie time), as well as ΔGmin(p-p) (minimum stimulating waveform peak-to-peak gradient excursion) and SRmin (minimum slew-rate needed to achieve stimulation). Note: The PNS threshold curves were obtained by simulation of trapezoidal waveforms with 500-μs plateau time and 16 bipolar cycles [file NIHMS1764395-supplement-Supplemental_figures.pdf]

Supplementary Material

A Huygens' surface approach to rapid characterization of Peripheral Nerve Stimulation

Mathias Davids[1,2,3,*], Bastien Guerin[1,2], Lawrence L. Wald[1,2,4]

[1]A.A. Martinos Center for Biomedical Imaging, Dept. of Radiology, Massachusetts General Hospital, Charlestown USA; [2]Harvard Medical School, Boston, Massachusetts, USA; [3]Computer Assisted Clinical Medicine, Medical Faculty Mannheim, Heidelberg University, Heidelberg, Germany; [4]Harvard-MIT Division of Health Sciences Technology, Cambridge, Massachusetts, USA

***Correspondence to mathias.davids@mgh.harvard.edu**

Simulation of Electric Fields

Generation of the P-matrix from a current basis set (either on the Huygens' surface or for any other geometry) requires an E-field simulation for each basis function. While EM simulations are typically truncated to the body regions under consideration, the Huygens' surface representation is more sensitive to truncation of the EM calculations. Truncation induces small errors of each Huygens' basis function that can accumulate constructively. Such errors can be uncovered when comparing field maps generated from the Huygens' surface representation to that of the full EM simulation. We, therefore, performed the field simulations in the full body model using an in-house low-frequency magneto quasi-static field solver based on the Modular Finite Element Methods (MFEM) C++ library. We implemented the standard eddy current problem where the E-field comprises a curl-free and a divergence-free term described by the electric scalar potential φ and magnetic vector potential $\mathbf{A}$**,** respectively:

$$\mathbf{E} = -\nabla\varphi + \frac{\mathrm{d}\mathbf{A}}{\mathrm{d}t} \quad . \qquad \text{Eq. 1}$$

The magnetic vector potential $\mathbf{A}$ is computed from a given current source $\mathbf{I}$ using the Biot-Savart formulation in integral form:

$$\boldsymbol{A}(\boldsymbol{r}) = \frac{\mu_0}{4\pi}\int_\Omega \frac{\boldsymbol{I}(\tilde{\boldsymbol{r}})}{\|\boldsymbol{r} - \tilde{\boldsymbol{r}}\|}\mathrm{d}^3\boldsymbol{r} \quad . \qquad \text{Eq. 2}$$

We then obtain the electric scalar potential φ by solving the differential form

$$\boldsymbol{A}\nabla \cdot \sigma\nabla\varphi = -i\omega\nabla \cdot \sigma\boldsymbol{A} \qquad \text{Eq. 3}$$

where σ is the spatially varying tissue conductivity. The electric scalar potential is modeled by a hexahedral finite element mesh (1 mm^3 cell size) yielding 76M mesh elements in our male model and 48.8M in the female model. Each cell is labeled by the tissue-specific conductivity obtained from the IT'IS LF database. We dramatically reduced computation time by taking advantage of the fact that the body model mesh is fixed for all Huygens' basis functions. This allowed us to reuse the FE system matrix on the left-hand side of Eq. 6 after the initial assembly (the most computationally intensive step). In our implementation, the E-field for each Huygens' basis can be computed in 2-3 minutes. The fast solver was validated by comparing the fields to the low-frequency solver in a commercial package (Sim4Life, Zurich MedTech).

PNS oracle calculation

The E-field of each basis function was projected onto the fibers of the body's nerve atlas. Integration along the nerve generated the electric potentials $V(r)$ along each of the $\sim$1900 nerves in the body. Using the pre-assigned axon diameter for each nerve, we computed the PNS oracle metric. The PNS oracle corresponds to the reciprocal of the PNS threshold coil current in units of Ampere, i.e., the PNS oracle has units of 1/A but can be easily related to the expected gradient strength at that current. The oracle estimates a nerve's PNS threshold based on the spatial characteristics of the electric potential $V(r)$ induced along the nerve path and the axon diameter of the nerve. The PNS oracle is defined by

$$\mathrm{PNSO}(r,D) = K(D) * \frac{V(r-L) - 2V(r) + V(r+L)}{L(D)^2} \cdot \frac{1}{m(D)} \qquad \text{Eq. 4}$$

where $L(D)$ is the node of Ranvier distance (which is a function of the axon diameter D), $K(D)$ is a spatial kernel, and $m(D)$ is the myelination calibration factor. The PNS oracle metric is based on the second spatial difference of the electric potential $V(r)$ across consecutive nodes of Ranvier (similar to previous definitions of the neural activating function and modified driving function). The result is convolved with a kernel $K(D)$ to describe current redistribution across nodes of Ranvier. The factor $m(D)$ accounts for the effect of different levels of myelination on the nerve's excitability. Both the spatial kernel $K(D)$ and the myelination factor $m(D)$ are calibrated for a specific coil current waveform: in this work we use a trapezoidal waveforms of varying rise times (500 µs flattop duration, 32 bipolar pulses). This calibration was done by comparing to the numerical titration of the double-cable nerve model in 64,000 nerve segments.

The oracle computation only uses operations linear in the applied current. This ensures that the PNS oracle for a coil configuration can be represented as a linear combination of the oracles of the individual basis functions using the same weights that would be used to formulate the coil configuration's B-field from its basis function B-fields. The linearity also allows the basis function PNS responses to be assembled into a matrix form (the *P*-matrix). Given a vector x listing the weights of the basis functions for

a specific winding pattern, the total PNS response and resulting PNS threshold can be quickly computed using

$$\boldsymbol{P}_{\text{total}} = \boldsymbol{P}x \qquad \text{and} \qquad \text{PNS threshold} = \frac{1}{\max\{|\boldsymbol{P}_{\text{total}}|\}} \qquad \text{Eq. 5}$$

This P-matrix product can be incorporated as a constraint in the gradient winding optimization or potentially used as a real-time safety watchdog during scanner operation.

Computational runtime

The Huygens' P-matrix computations fall into two categories, computationally intensive steps intended to be performed once and computationally efficient steps intended to be performed for each coil geometry and patient position. The computationally intensive steps included the pre-calculation step of the Huygens' P-matrix which consists of EM calculations and compilation of the PNS oracles into the P-matrix. These steps were performed on a high-end Linux server (using 300 GB of memory and 20 cores), taking 3-5 days per body model. The simpler calculations are expected to be done multiple times (e.g. for every body position) and were done a high-end laptop (using 8 GB of memory and 6 cores). These simpler steps included computing the mapping matrix M for a given body position, application of this matrix to the Huygens' P-matrix and evaluation of PNS thresholds by multiplying the resulting P-matrix by the current weight vector describing the coil configuration. Evaluating PNS for a given coil winding pattern is fast (~5 seconds) since in this case the Huygens' basis set is mapped to a single "excitation source", i.e., a single gradient coil. The mapping matrix calculation required for coil winding optimization is more expensive, as it requires a mapping of the Huygens' basis set to the thousands of "excitation sources", i.e., the current density elements on the coil former (7595 in our example), requiring ~1 minute. Evaluation of PNS thresholds for a given trial current basis set is a very fast matrix-multiply of the coil geometry specific P-matrix PC with the candidate basis vector (requiring milliseconds).

The FEM electromagnetic simulations constitute by far the most expensive step of the precomputation phase. As previously described, these field simulations require relatively high spatial resolution (~1 mm3) to correctly capture local E-field hotspots frequently occurring, e.g., in thin layers of fatty tissue through which most nerves run in the body. This leads to large FEM simulation domains with up to 76M mesh cells. Additionally, the body model simulation domain cannot be truncated to reduce computation time. We found, that truncation of the simulation domain (e.g., excluding portions of the model with negligible B-field amplitudes) led to unpredictable constructive or destructive error propagation and incorrect PNS thresholds. We dealt with the additional computational demand incurred in the whole-body simulations by developing our own in-house EM field solver based on the lightweight, heavily parallelized MFEM C++ library. This allowed us to reduce computation time from ~45 minutes to 2-3 minutes per Huygens' basis.

Gradient coil	$\Delta G_{min(0\text{-}p)}$ [mT/m]	t_{chron} [µs]	$\Delta G_{min(p\text{-}p)}$ [mT/m]	SR_{min} [T/m/s]
**X**-axis, no PNS opt.	36.9	413	73.8	178.7
**X**-axis, with PNS opt.	52.8	377	105.6	280.1
**Y**-axis, no PNS opt.	14.0	395	28.0	70.9
**Y**-axis, with PNS opt.	27.4	411	54.8	133.3
**Z**-axis, no PNS opt.	23.5	395	47.0	119.0
**Z**-axis, with PNS opt.	32.2	439	64.4	146.7

Supplementary Table S1: Summary of PNS threshold curve parameters $\Delta G_{min(0\text{-}p)}$ (minimum stimulating waveform zero-to-peak gradient amplitude), t_{chron} (chronaxie time), as well as $\Delta G_{min(p\text{-}p)}$ (minimum stimulating waveform peak-to-peak gradient excursion) and SR_{min} (minimum slew-rate needed to achieve stimulation). The PNS threshold curves were obtained by simulation of trapezoidal waveforms with 500 µs plateau time and 16 bipolar cycles.

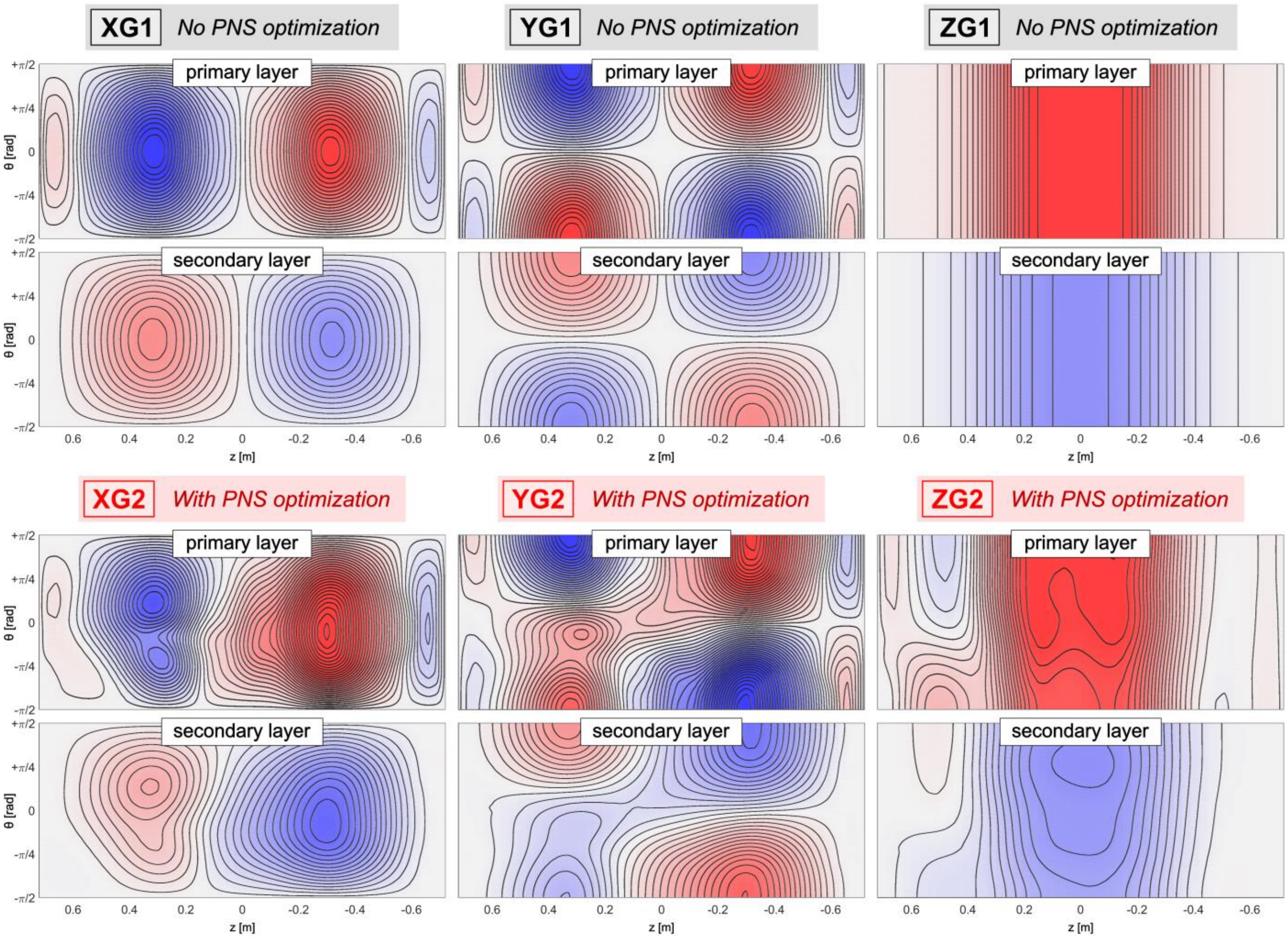

Supplementary Figure S1: Unfolded winding patterns for the unoptimized and optimized body gradient coils. The coils were designed using our PNS constrained BEM-SF optimization framework published recently and were optimized using our male body model for head-imaging using a head-first supine body position. The increase in coil inductance for the PNS optimized coils (XG2, YG2, ZG2) was restricted to 15% compared to the coils without PNS optimization (XG1, YG1, ZG1).
